# Searching for dark matter at colliders

**DOI:** 10.1140/epjc/s10052-015-3379-8

**Published:** 2015-04-28

**Authors:** Francois Richard, Giorgio Arcadi, Yann Mambrini

**Affiliations:** Laboratoire de l’Accélérateur Linéaire, IN2P3/CNRS and Université Paris-Sud 11 Centre Scientifique d’Orsay, B. P. 34, 91898 Orsay Cedex, France; Laboratoire de Physique Théorique, Université Paris-Sud, 91405 Orsay, France

## Abstract

Dark Matter (DM) detection prospects at future $e^+e^-$ colliders are reviewed under the assumption that DM particles are fermions of the Majorana or Dirac type. Although the discussion is quite general, one will keep in mind the recently proposed candidate based on an excess of energetic photons observed in the center of our Galaxy with the Fermi-LAT satellite. In the first part we will assume that DM interactions are mediated by vector bosons, $Z$ or $Z'$. In the case of $Z$-boson Direct Detection limits force only axial couplings with the DM. This solution can be naturally accommodated by Majorana DM but is disfavored by the GC excess. Viable scenarios can be instead found in the case of $Z'$ mediator. These scenarios can be tested at $e^{+}e^{-}$ colliders through ISR events, $e^+e^-\rightarrow XX+\gamma$. A sensitive background reduction can be achieved by using highly polarized beams. In the second part scalar particles, in particular Higgs particles, have been considered as mediators. The case of the SM Higgs mediator is excluded by limits on the invisible branching ratio of the Higgs. On the contrary particularly interesting is the case in which the DM interactions are mediated by the pseudoscalar state $A$ in two Higgs-doublet model scenarios. In this last case the main collider signature is $e^+e^-\rightarrow HA, H\rightarrow hh, A \rightarrow XX$.

## Introduction

The search for dark matter is of prime importance for our understanding of the universe. This goal is pursued using a wide variety of approaches, given the very large spectrum of interpretations predicting particles with a mass range between $\upmu$ev, multi TeV and even beyond, from axions to wimpzillas.

On recent times several hints for detection have come from Indirect Dark Matter Detection (ID) searches, i.e. lines at 3.5 KeV [1, 2] and 130 GeV [3, 4] and the $\gamma$-ray excess from the galactic center [5]. No consistent picture emerges so far. Moreover, this kind of signal can be attributed to astrophysical sources or instrumental effects [6, 7].

Similarly previous hints in Direct Detection (DD) seem contradicted by recent results by SuperCDMS [8], XENON100 [9] and LUX [10], unless rather particular scenarios are assumed (see e.g. [11, 12]). These experiments are reaching increasingly higher sensitivities and are thus capable to probe a very broad range of models.

Collider searches are therefore the necessary complement for a safe conclusion on this essential investigation. Here we will focus on the prospects offered by future $e^+e^-$ colliders (see also [13–17] for similar studies), in particular the International Linear Collider, ILC, with polarized beams, in testing DM scenarios providing an interpretation of the recently reported GC gamma-ray excess.

For our investigation we will consider simplified scenarios (see also [18–28] for similar approaches) in which a fermionic (Dirac or Majorana) WIMP DM, with mass in the range $30$–$50\,\text{ GeV }$, favored by the GC signal, is coupled either with SM mediators, namely the $Z$ boson or the Higgs, or with BSM mediators, namely $Z^{'}$, scalars and pseudoscalar states from an extended Higgs sector. We will also apply to these scenarios the constraints from LUX, the invisible *Z* width from LEP1 and the invisible H width from LHC. We also remark that direct production at linear colliders of the mediators allows for the observation of distinctive signatures, in the form of resonances, and to profit of the optimal capability of mass reconstruction of these kind of colliders.

The paper is organized as follows. In Sect. 2 we will summarize the main information regarding the Galactic Center (GC) excess and state the kind of scenarios which we are going to analyze. Section 3 and 4 will then be dedicated, respectively, to $Z$ and $Z^{'}$ portal scenarios. After a brief review of the SM Higgs portal case we will investigate two cases of extensions of the Higgs sector, namely the addition of a scalar and a pseudoscalar Higgs singlet and a two Higgs-doublet scenario. After this we will state our conclusions.

## The galactic center photon excess

A recent study [5] has reported the existence of $\gamma$-ray excess from the GC which could be interpreted as the signal of the annihilation into $\bar{b} b$ final states of a DM with mass of approximately 35 GeV or the democratic annihilation into SM fermions of a 25 GeV mass DM. In both cases the required value of the DM annihilation cross section is of the order of the cosmologically favored value $\langle \sigma v \rangle \sim 3 \times 10^{-26} {\text{ cm }}^3/s$ [29]. A more recent thorough analysis [30] has confirmed this excess but favoring a slightly higher value of the DM mass, $49 \pm 6$ GeV.

This annihilation process can be interpreted through several combinations of DM particles and mediators (see e.g. [31] for a rather extensive classification).

In the case of SM mediators, like the $Z$ and the Higgs, such low values of the DM mass require one to take into account existing accelerator limits on the invisible decay of these particles. Moreover, it is necessary to cope with the strong limits provided by the LUX experiment for spin-independent (SI) interactions, which reaches its full sensitivity in the mass region claimed for the Fermi-LAT signal. Recall, however, that the SI cross section limits assume coherent recoil of the nucleus caused by the DM scattering. For heavy nuclear targets, the coherent scattering increases the cross section by the square of the atomic number. This is not the case for the spin-dependent (SD) cross section, which occurs through coupling to the spin content of the nucleus, meaning that the cross-section limits are about four orders of magnitude weaker than for SI. As a consequence sensitively weaker constraints would be obtained in the case in which the DM features only spin-dependent interactions with the nucleon. This is, for example, the case of a Majorana DM coupled with a $Z/Z^{'}$ mediator. In this case, indeed, the only possible coupling is axial, which induces only the spin-dependent component in the DM scattering cross section.

This choice, however, leads to a velocity-dependent pair annihilation cross section. In such a case it is not possible to achieve at the same time the correct DM relic density and reproduce the GC signal because of the very different values of the DM velocity at the time of decoupling and at present times. A possible way out could be provided by the presence of extra interactions leading to a Sommerfeld enhancement of the annihilation cross section at low velocities [32–34].

In $Z^{'}$ scenarios it is possible to have suppressed DD cross section also for DM by choosing a suitable combination of the couplings. For example, it is possible to assume that the DM is coupled only vectorially with the $Z^{'}$ and the latter only axially coupled with SM fermions.

Alternatively one can assume that the DM is axially coupled with scalar/pseudoscalar mediators. The case of the SM is already excluded by LHC limits since, in order to reproduce the GC excess, a too large invisible decay width of the Higgs is needed. An extended Higgs sector can provide instead viable solutions.

We remark that in all the mentioned scenarios, in particular the ones with BSM mediators, the dark matter candidate is assumed to be lighter than the mediator of its interactions with the Standard model states. If this is not the case $2 \rightarrow 2$, or even $2 \rightarrow 3$, production of on-shell vector or scalar/pseudoscalar states, with the latter subsequently decaying into $b \bar{b}$ pairs, may be relevant and possibly account for the GC signal. This scenario is extensively discussed e.g. in [35] and it is shown that DM candidates with masses above 100 GeV are favored. As already mentioned we will focus, in this work, on lighter dark matter candidates and we will implicitly assume, unless explicitly stated, that the production of mediators from dark matter annihilations is kinematically forbidden.

We report in Table 1 three scenarios which can be hardly probed by DM direct detection: Majorana DM + $Z^{'}$ mediator, Dirac DM with vectorial coupling to a $Z^{'}$ only axially coupled to SM fermions and Majorana DM coupled to a pseudoscalar mediator. On the contrary these scenarios can be covered by LHC searches and, as will be discussed below, complementary information can be provided by $e^+ e^-$ colliders.

**Table 1:** Summary of main scenarios considered in this work

DM	Mediator	Interaction	Direct	LHC
Majorana	$Z/Z^{'}$	$\bar{X}\gamma ^\mu \gamma _5 X$,$\bar{f}\gamma ^\mu \gamma _5 f$	Yes	Yes
Dirac	$Z^{'}$	$\bar{X}\gamma ^\mu X$,$\bar{f}\gamma ^\mu \gamma _5 f$	No	Yes
Majorana	$A$	$\bar{X}\gamma _5 X$,$\bar{f} \gamma _5 f$	No	Yes

## *Z* portal

### Thermal freeze-out

For masses in the range relevant for the GC signal the DM annihilates into fermion pairs through *Z*-boson exchange in the s channel. This process is described by the following lagrangian:

1 $$\begin{aligned} \mathcal {L}&=\left[ a \bar{X}\gamma ^\mu \left( g_V^X +g_A^X \gamma _5 \right) X\right] Z_\mu \nonumber \\&\quad + \left[ \bar{f}\gamma ^\mu \left( g_V^f +g_A^f \gamma _5 \right) f\right] Z_\mu \end{aligned}$$

where $a=1(1/2)$ for Dirac (Majorana) DM (In the case of Majorana DM $g_V^X=0$.). As shown in [36] the value of $g_V^X$ should be extremely suppressed because of the limits from LUX. For simplicity we will thus focus on the case of a Majorana DM. Neglecting fermion masses, at decoupling one has

2 $$\begin{aligned} \begin{aligned}&\sigma v= \sum _f n_\mathrm{cf} \left( \left| g_V^f\right| ^2+\left| g_A^f\right| ^2\right) \\&\frac{\left| g_A^X\right| ^2 s v^2}{12\pi \left[ {\left( s-m_Z^2\right) }^2+m_Z^2 \Gamma _Z^2\right] } \end{aligned} \end{aligned}$$

where $g_V^f=\frac{g}{2 c_W}\left( I_3 -2 Q_f s_W^2\right) ,\ g_A^f=\frac{g}{2 c_W}I_3$, and the $v^2 \sim 0.24$. Notice that this expression is valid only away from the $Z$-pole, $m_\chi \sim m_Z/2$, where one should rely on a more refined computation [37, 38].

**Fig. 1:**
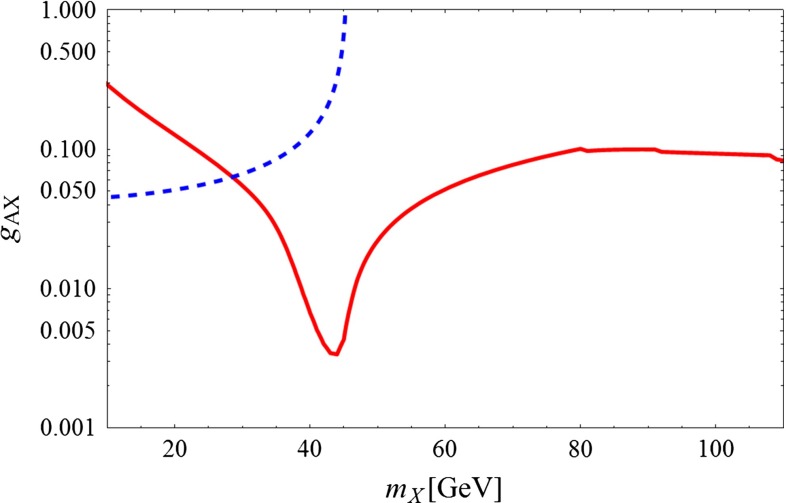
Predicted axial coupling of *Z* to Majorana DM fermions versus their mass. The *blue dashed curve* comes from the *Z* invisible width limit from LEP1

We show in Fig. 1, the dependence of the axial coupling with respect to $m_X$. This curve, which relies on the exact expression of the annihilation cross section, without performing the velocity expansion, differs appreciably, up to a factor 2 at resonance, from our analytical estimation. This detail is of importance since the GC signal favors DM masses close to the resonance.

### Annihilation signal from the galactic center

After decoupling, our universe cooled down and, at present, the velocity of DM is $\sim$300 km/s, that is, $v=0.001$. This means that the annihilation cross section previously computed becomes completely negligible and therefore unable to explain the indirect signal observed by Fermi-LAT. Note, however, that our calculation of the annihilation cross section has neglected the $s$-wave contribution proportional to the fermion masses; this is not legitimate for the b quark when $v\rightarrow 0$. Taking into account this term we can relate the annihilation cross section at present times with the one at freeze-out as

3 $$\begin{aligned} \frac{\sigma v_\mathrm{GC}}{\sigma v_\mathrm{FO}}=3 \mathrm{Br}(Z \rightarrow \bar{b} b) \frac{m_b^2}{s v^2}\left[ 1-\frac{s}{m_Z^2}\right] ^2 \end{aligned}$$

At present times the DM annihilates dominantly into $b \bar{b}$, thus satisfying [5]; however, the annihilation cross section is $O(10^3)$ times smaller than the value at freeze-out.

The $Z$-portal scenario thus appears to be inconsistent with the GC excess. We will nonetheless retain it, assuming the presence of a low velocity Sommerfeld enhancement of the annihilation cross section.

### The *Z* invisible width and the ISR measurement.

The *Z* invisible width has been very precisely measured at LEP1 and can be modified if there is a substantial decay of *Z* into *X* Majorana fermions. One has

4 $$\begin{aligned} \Gamma (Z \rightarrow \bar{X} X)=\frac{|g_A^X|^2 v^3 m_Z}{24 \pi } \end{aligned}$$

where

5 $$\begin{aligned} v=\sqrt{1-\frac{4 m_X^2}{m_Z^2}}. \end{aligned}$$

Using the upper limit of 2 MeV for the BSM invisible by LEP1, one can exclude solutions with $m_X<27$ GeV, which is compatible with the interpretation given in [5]. For a Dirac fermion, with an axial coupling, one has $m_X<29$ GeV.

At future $e^+e^-$ colliders, a factor of $\sim$2 in precision appears feasible taking into account the dominant contribution due to luminosity accuracy at $0.1\,\%$. This gives $m_X<28.5$ GeV for a Majorana fermion.

An alternative method uses radiative return to the *Z* peak by running at a circular collider [39] above this peak, at maximum integrated luminosity. Systematical errors represent a limitation, however, as argued by [39], using the leptonic modes for normalization, one can remove most uncertainties. This approach could achieve up to an order of magnitude accuracy improvement. Even then, the invisible *Z* width method can only cover masses up to 35 GeV.

In the *Z* portal scenario, LEP results can exclude a Majorana fermion with mass below $m_X=27$ GeV, insufficient to interpret/exclude the GC photon excess. Future $e^+e^-$ colliders will reach at best $m_X=35$ GeV. For what concerns LHC, given the predicted branching ratio of *Z* into DM, no observable signal can be seen by the monojet search above the large background due to *Z* decays into neutrinos.

## $Z^{'}$ portal

As we shall see, a $Z^{'}$ scenario offers many opportunities thanks to additional free-parameters, with respect to the $Z$ mediator case, consisting in the mass $m_{Z^{'}}$ and the couplings, vectorial and axial, of the $Z^{'}$ with the SM fermions. The relevant interactions are described by an analogous lagrangian as the $Z$ case:

6 $$\begin{aligned} \mathcal {L}&=\left[ a \bar{X}\gamma ^\mu \left( g_V^X +g_A^X \gamma _5 \right) X\right] Z^{'}_\mu \nonumber \\&\quad + \left[ \bar{f}\gamma ^\mu \left( \tilde{g}_V^f +\tilde{g}_A^f \gamma _5 \right) f\right] Z^{'}_\mu \end{aligned}$$

In order to keep the discussion as general as possible we will consider a generic parameterization for the new couplings $\tilde{g}_V^f$ and $\tilde{g}_A^f$ normalizing them to the corresponding coupling of the $Z$-boson, i.e. $\tilde{g}_{V,A}^f=K g_{V,A}$, where $g_V^f$ and $g_A^f$ have been defined in the previous section, or eventually setting some of them to zero in order to comply with experimental constraints.^1^ In the following we will envisage two scenarios:

- **Scenario 1**: A $Z{'}$ axially coupled with ordinary matter and vectorially coupled with a Dirac DM.
- **Scenario 2**: A $Z{'}$ axially coupled with a Majorana DM and with both vectorial and axial couplings with ordinary matter.

### Scenario 1

This scenario corresponds to the assignment $a=1$, $g_V^X=0$, $\tilde{g}_{V}^f=K g_{V}^f$, and $\tilde{g}_A^f=0$. The pair annihilation cross section features an unsuppressed s-wave contribution of the form:

7 $$\begin{aligned}&\sigma v= |g_V^X|^2 K^2 \sum _f n_\mathrm{cf} |g_A^f|^2 \nonumber \\&\frac{s+2 m_X^2}{12\pi \left[ {\left( s-m_{Z^{'}}^2\right) }^2+m_Z^2 \Gamma _{Z^{'}}^2\right] } \end{aligned}$$

and it is thus possible to reproduce the GC signal without invoking a Sommerfeld enhancement. In absence of vectorial couplings of the $Z^{'}$ with SM fermions the SI scattering cross section is heavily suppressed and thus does not affect phenomenology. As already mentioned before we will focus on the case of a DM pair annihilation into SM fermions through s-channel mediation of a $Z^{'}$ state heavier with respect to it. We just mention that it is possible to obtain comparable values, of the order to the cosmologically favored one, for the total DM cross section at present times and at decoupling also for a DM mass of the order of 80–100 GeV and a rather light $Z^{'}$, with mass of the order of 30 GeV and for $K \sim 0.1$. In such a case the relevant contribution is given by the $XX \rightarrow Z^{'} Z^{'}$ annihilation. This kind of scenario requires, however, a dedicated study and will not be further considered in this work.

### Scenario 2

Similarly to the $Z$-portal scenario one can consider a DM only axially coupled with the $Z^{'}$ [40] which, in turn, features both vectorial and axial couplings with SM fermions. This is naturally realized in the case of Majorana DM. The annihilation cross section is velocity dependent and given by

8 $$\begin{aligned}&\sigma v= \left| g_A^X\right| ^2 K^2 \sum _f n_\mathrm{cf} \left( \left| g_V^f\right| ^2+\left| g_A^f\right| ^2\right) \nonumber \\&\frac{s v^2}{\left[ {\left( s-m_{Z^{'}}^2\right) }^2+m_{Z^{'}}^2 \Gamma _{Z^{'}}^2\right] } \end{aligned}$$

As a consequence, it is possible to reproduce the GC signal only in presence of a Sommerfeld enhancement.

Contrary to scenario 1 the case $m_X > m_{Z^{'}}$ does not provide viable solutions. Indeed in this case the coupling $g_A^X$ can be constrained by limits from the LUX experiment on the SD cross section [36], which for low masses of the mediator are strong enough to exclude thermal values for the annihilation cross section of the DM into two $Z^{'}$.

As will be explained in the next subsection, in order to comply with current LHC limits, it is necessary, in both scenarios, to have a $Z^{'}$ decaying mostly invisibly, which requires using the ISR technique in $e^+e^-$. We will briefly review, in the next subsection, this technique for what concerns the ILC setup and describe the prospects of discovery for a $Z^{'}$ scenario (see also [41] for an analogous description relative to LEP).

### The ISR approach at ILC

**Fig. 2:**
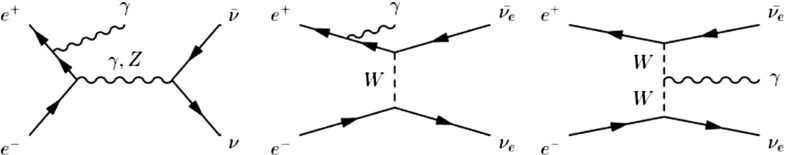
Relevant diagrams for $e^+ e^- \rightarrow \nu _e \nu _e \gamma$ background process

To probe a heavy invisible $Z^{'}$ scenario, one needs to operate at high energies and use initial state radiation (ISR) at angle. Above the *Z* pole, the main background comes from $e^+e^- \rightarrow \nu _e \nu _e \gamma$ with W exchange (see the diagrams in Fig. 2). This process is only sensitive to left handed electrons and therefore can be efficiently removed using right handed polarization for electrons, which can be provided by ILC [42].

One can also assume an improved polarization [43] with respect to the base line ILC parameters: $Pe-=90\,\%$ and $Pe+=-60\,\%$ instead of $Pe-=80\,\%$ and $Pe+=-30\,\%$. The corresponding suppression of the W exchange process improves by a factor 4. To understand this effect, recall that this suppression goes like $1-P$, where $P=(Pe-+Pe+)/(1-Pe-Pe+)$ is the effective polarization. There should be a negligible contamination due to $e^+e^- \rightarrow e^+e^- \gamma$. This assumption is detector and machine dependent and requires more work to be established. In principle, it is possible to eliminate this background by requesting a photon with sufficient transverse momentum, which guarantees the appearance of an energetic $e^+/e^-$ in the forward electromagnetic calorimeters. This demands perfect vetoing of electrons in these calorimeters which is only possible if the beam background remains at a manageable level. A careful optimization of the final focus region is needed to avoid overloading the very forward calorimeters (see for instance reference [44, 45]).

A heavy $Z^{'}$ requires a large coupling to DM and therefore a wide resonance decaying mostly invisible. This type of scenario has already been discussed in [46]. If the $Z^{'}$ couplings to standard fermions are not suppressed with respect to the SM, the limits set by ATLAS/CMS for lepton pairs are still able to exclude this solution. Assuming a reduction factor $K^2 \sim 0.1$ on the standard couplings allows one to reach a wide domain of solutions as discussed below.

In scenario 1 the width of the $Z^{'}$ is given by

9 $$\begin{aligned} \Gamma (Z^{'} \rightarrow \bar{X} X)=\frac{|g_V^X|^2 v m_{Z^{'}}}{12 \pi } \end{aligned}$$

**Fig. 3:**
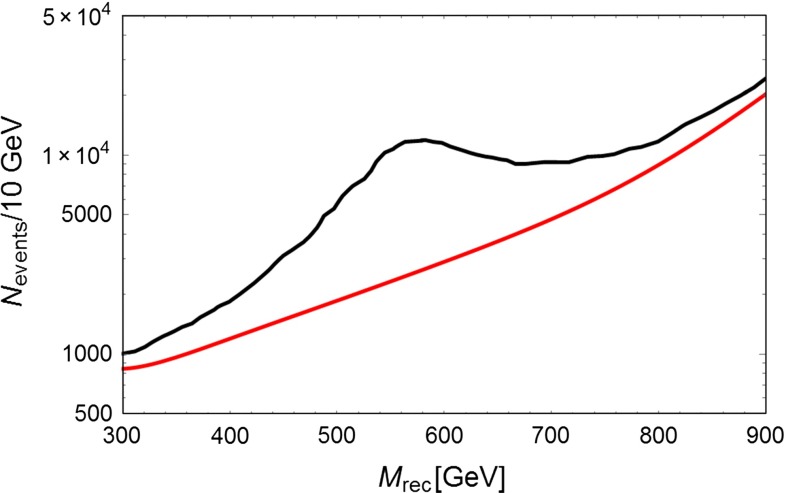
Number of expected ISR events in 10 GeV bins versus the effective center of mass energy. The *red curve* shows the expected background. The *black curve* is the predicted rate assuming a $Z^{'}$ with $m_{Z^{'}}=550$ GeV. These curves correspond to an ILC operated at 1 TeV and collecting $1 {\text{ ab }}^{-1}$ with beam polarizations $Pe-=0.9$ and $Pe+=-0.6$

**Fig. 4:**
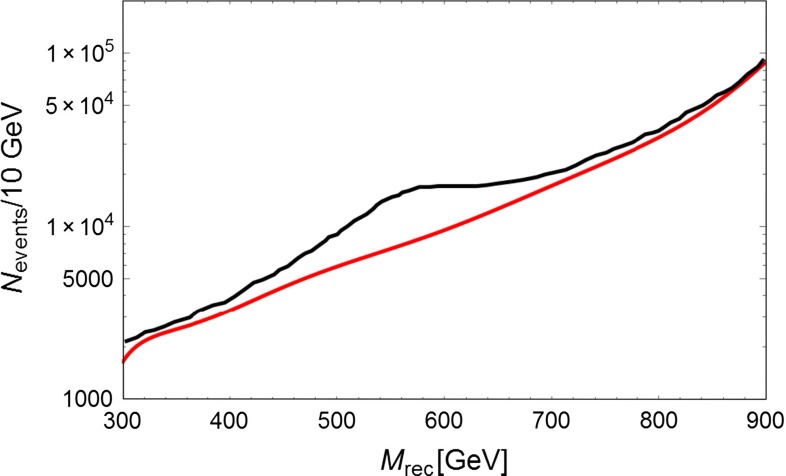
*Right panel* The same as Fig. 3 but with $Pe-=0.8$ and $Pe+=-0.3$

As already mentioned, one can use an ISR method requesting a photon emitted inside the detector. Measuring its energy $k$, one can determine the recoil mass from the expression: where $E_\mathrm{CM}$ is the center of mass energy and $x=2k/E_\mathrm{CM}$. One can assume $m_{Z^{'}}=550$ GeV and an ILC operating at $E_\mathrm{CM}=1$ TeV at full luminosity $(1 ab^{-1}$) and with improved polarization, as previously defined. Figure 3 shows that, as expected, $M_\mathrm{rec}$ peaks at the $Z^{'}$ mass. The fast rising background is due to $W$ exchange. The significance of the signal is very high since at the resonance one counts about 10,000 events per 10 GeV bin with an expected background of $\sim$2000 events. Figure 4 considers more conservative assumptions on beam polarization ($Pe-=80\,\%$ and $Pe+=-30\,\%$) and shows that a signal excess is still observable.

It should be underlined that this invisible $Z^{'}$ scenario is uniquely covered with this ISR method at ILC and could escape to direct observation into lepton pairs at LHC.

From Fig. 2, one concludes that this method allows one to measure the $Z^{'}$ mass, its total width $\Gamma _t \sim \Gamma _\mathrm{inv}$ and its invisible cross section at resonance $\sigma \sim \mathrm{BR}_\mathrm{ee} \mathrm{BR}_\mathrm{inv}$ with $\mathrm{BR}_\mathrm{inv} \sim 1$. From these two measurements, one extracts the couplings of $Z^{\prime }$ to DM and to $e^+e^-$, which allows one to draw some important clues about the underlying model.

### Comparison with LHC

**Fig. 5:**
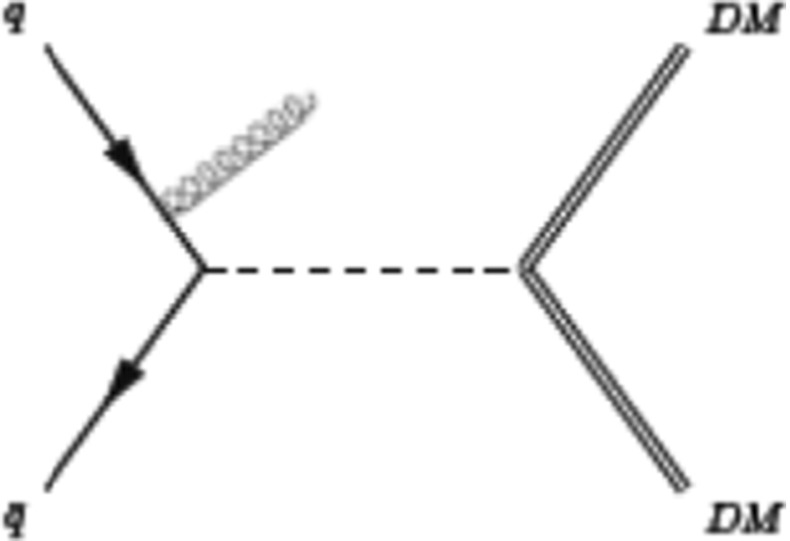
Example of diagram contribution to DM pair+ monojet production

A quasi-invisible $Z^{'}$ could, in principle, also be observed at LHC using the monojet technique (see e.g. [47–52]), as illustrated by the diagram in Fig. 5 which is of course analogous to the ISR technique in $e^+e^-$. To compare the two types of colliders, one notes that, at LHC, the invisible $Z^{'}$ cross section goes like $K^2 g^2_\mathrm{Zqq} \mathrm{BR}_{Z^{'}\,\mathrm{inv}}$ with $\mathrm{BR}_{Z^{'}\,\mathrm{inv}} \sim 1$. Since $\mathrm{BR}_{Z\,\mathrm{inv}}=0.2$, the rate is reduced by $5K^2 \mathrm{RL}$ with respect to the inclusive production of invisible *Z* where RL is a luminosity ratio which takes care of the difference of mass between $Z$ and $Z^{'}$. This ratio tends to 1 when $m_{Z^{'}} \ll E_{t,\mathrm{miss}}$, where $E_{t,\mathrm{miss}}$ is the transverse energy carried by the gluon. With the most recent data [53, 54] taken at 8 TeV, the sensitivity of LHC is reaching $E_{t,\mathrm{miss}} \sim 800$ GeV. For $m_{Z^{'}}= 500$ GeV and $E_{t,\mathrm{miss}}=800$ GeV, one has $\mathrm{RL} \sim 0.6$. The expected excess over the *Z* contribution would be $\sim$+30 %, barely observable. LHC at 14 TeV will allow one to reach $m_{Z^{'}}=1$ TeV. Clearly these conclusions may be moved around by modifying the parameter $K^2$.

**Fig. 6:**
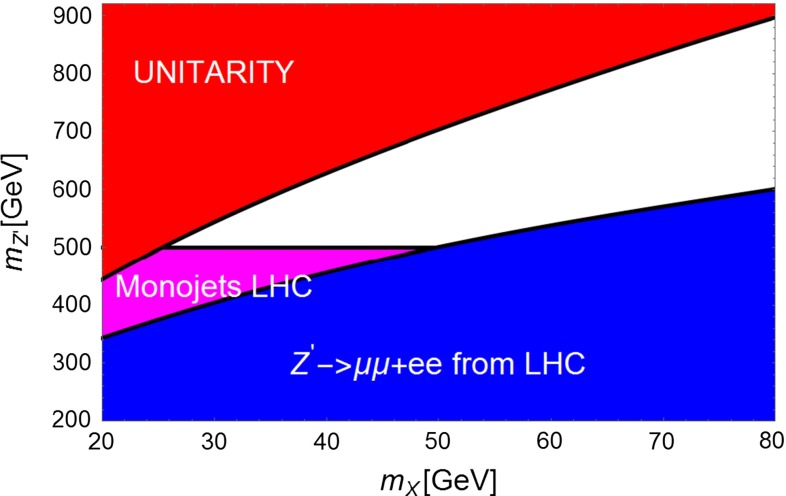
Summary plot for scenario 1 for $K=0.1$. The *red region* corresponds to coupling of the DM with the $Z^{'}$ exceeding unitarity limit. The *blue region* is excluded by LHC searches of dilepton resonances. The *magenta region* would correspond to a visible excess of monojet plus missing energy events

**Fig. 7:**
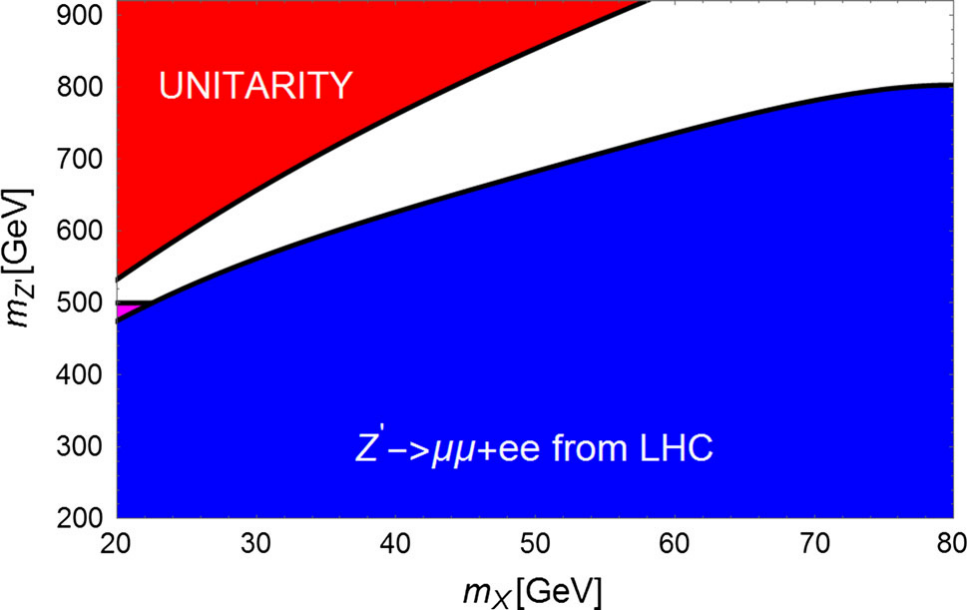
The same as Fig. 3 but for $K^2=0.2$

Figures 6 and 7 summarize, fixing $K^2=0.1$, the mass regions already excluded by present searches of $Z^{'}$ into lepton pairs (blue) and indicates the region sensitive to monojets. The red area is excluded by the unitarity limit, $g_A^{X\,2} <4\pi$. For $m_X \sim 35\,\text{ GeV }$ this scenario will be probed by LHC in the next future.

If LHC observes only an excess at large $E_{t,\mathrm{miss}}$ and no lepton pair signal, the interpretation of such a signal would be uncertain since, without the initial state energy-momentum constraint, one cannot observe the resonance shape shown in Figs. 3 and 4. ILC operating at 1 TeV and collecting $1 ab^{-1}$ can fully cover the white allowed area and allow for the reconstruction of the $Z^{'}$ mass in case of a signal.

In conclusion, for the invisible $Z^{'}$ scenario, ILC provides a unique opportunity of detection based on radiative return and background suppression using highly polarized beams. Within the Fermi-LAT scenario, there are good prospects of discovery at LHC for this scenario.

In scenario 2, the DM being a Majorana fermion, the decay width of the $Z^{'}$ reads

10 $$\begin{aligned} \Gamma (Z^{'} \rightarrow \bar{X} X)=\frac{|g_A^X|^2 v^3 m_{Z^{'}}}{24 \pi }. \end{aligned}$$

The previous analysis can be anyway carried out along the same steps leading to similar conclusions as scenario 1.

## SM Higgs portal

An interaction between a SM singlet fermionic DM and the SM Higgs can be achieved, contrary the case of a scalar DM (see e.g. [55] for a recent update in experimental constraints), by means of the following dimension-5 operators:

11 $$\begin{aligned} \mathcal {L} \supset H^{\dagger } H \left[ \frac{\lambda _S^X}{\Lambda } \bar{X} X + i \frac{\lambda _P^X}{\Lambda } \bar{X} \gamma _5 X\right] \end{aligned}$$

where $\Lambda$ represents a generic scale of new physics. Scalar type interactions are, however, essentially excluded by DM Direct Detection [56–60]; for simplicity we will thus set $\lambda _S^X=0$. Since the couplings of the Higgs bosons with SM fermions are proportional to the mass of the fermion themselves the main annihilation channel is into $\bar{b} b$. The cross section is given by

12 $$\begin{aligned} \sigma v=\frac{3 m_b^2 |\lambda _P^X|^2 s}{16 \pi v_h^2 {\left( s-m_h^2\right) }^2} \end{aligned}$$

where we have not included the Higgs width in the propagator, since we are focusing on DM masses in the range 30–50 GeV, compatible with the GC excess, still far enough from resonance, and we have performed the rescaling $\lambda _P^X \rightarrow \frac{v_h}{\Lambda } \lambda _P^X$ with $v_h$ being the v.e.v. of the Higgs. The thermal value of the cross section is achieved for $\lambda _P^X \approx 2.4$. This same coupling, however, determines the invisible width of the Higgs:

13 $$\begin{aligned} \Gamma _{h,\, \mathrm{inv}}=\Gamma (h \rightarrow \bar{X} X)=\frac{|\lambda _P^X|^2 v^3 m_h}{16 \pi }. \end{aligned}$$

For the value of the parameter $\lambda _P^X$ satisfying the GC excess we have $\Gamma _{h,\,\mathrm{inv}}=8\,\text{ GeV }$, which largely exceeds the SM expectation of 4 MeV of the total width of the Higgs. As a consequence the SM Higgs portal cannot explain the Fermi-LAT excess.

It is anyway worth investigating whether $e^{+}e^{-}$ can probe higher values of the DM mass, above the kinematic threshold of the Higgs decay, possibly renouncing to the explanation of the GC excess.

As the DM mass increases, the coupling to the DM should rapidly vanish in order to comply with the correct DM relic density. For $m_X$ above 60 GeV the invisible branching fraction goes below $10\,\%$ and therefore can only be excluded at $e^+e^-$ colliders by using the $Zh$ mode, with Z decaying into lepton pairs, which allows for a precise recoil mass reconstruction. In this way, $e^+e^-$ machines can provide a model-independent measurement of the invisible Higgs width and one can measure the invisible branching ratio down to a $\%$ level [61].

If $m_X>m_h/2$ one can still produce a virtual $h^*$, but the cross section decreases rapidly and the increase in mass coverage is marginal as reported in [62]. Here it has been envisaged the possibility to use the fusion process $e^+ e^- \rightarrow ZZ e^+ e^-$ with $ZZ\rightarrow h^*$. When $h^*$ decays invisibly, it is still possible to reduce the backgrounds by using the final state leptons. At 3 TeV center of mass energy, reachable by CLIC, the increase in mass coverage is also marginal with the predicted Higgs DM couplings.

## Higgs singlets

We now consider the case that the main interactions of the DM are with two Higgs singlets, $s$ and $a$, being, respectively, a scalar and a pseudoscalar state, also interacting with SM fermions according to the following lagrangian:

14 $$\begin{aligned} \mathcal {L}&= s \left[ \lambda _s^X \bar{X} X + \lambda _s^f \bar{f} f\right] + \left[ i \lambda _a^X \bar{X} \gamma _5 X+ i \lambda _a^f \bar{f} \gamma _5 f \right] \end{aligned}$$

The DM can annihilate into fermion pairs through s-channel mediation of the s/a fields. The corresponding cross section reads

15 $$\begin{aligned} \sigma v&=\frac{3 |\lambda _a^b \lambda _a^X|^2 s}{32 \pi {\left( s-m_a^2\right) }^2}+\frac{3 s |\lambda _s^b \lambda _s^X|^2 v^2}{64 \pi {\left( s-m_s^2\right) }^2}\nonumber \\&\quad +\frac{3s \left( \lambda _a^b \lambda _s^b \lambda _a^X \lambda _s^X v^2\right) }{64 \pi \left( s-m_a^2\right) \left( s-m_s^2\right) }. \end{aligned}$$

In order to account for the GC signal we assume that the couplings of the new states with SM fermions have a similar structure as the ones of the Higgs couplings, i.e. $\lambda _s^f = c_s \frac{m_f}{v_h}, \lambda _a^f=c_a \frac{m_f}{v_h}$. For simplicity we will also assume $\lambda _s^X=\lambda _a^X$.

As we notice the contribution from pure pseudoscalar mediation is not velocity suppressed. It is then possible to fit the GC signal in case this is the dominant contribution. This requirement is actually rather easy to fulfill since the couplings of the pseudoscalar are substantially irrelevant for DM direct detection [31] while, on the contrary, scalar couplings are very severely constrained by LUX limits. Under our assumptions these translates into the requirement $m_s \gg m_a$. A good fit of the GC signal is provided, for example, for a DM mass $m_X=35\,\text{ GeV }$, $m_a=50\text{ GeV }$, $\lambda _a^b=0.01$ (corresponding to the value of the corresponding SM Higgs coupling) and $\lambda _a^X=0.5$. This solution is very similar to the one obtained in NMSSM setups [65–67]. There is actually a slight mismatch between the annihilation cross section at freeze-out and at present time because of the thermal broadening [37, 38] of the width of the pseudoscalar. This mismatch can be solved by increasing the cross section at freeze-out, e.g. by adding an axial coupling between the DM and the $Z$ boson. Indeed, as pointed out before, s-channel interactions with an axially coupled $Z$-boson would change DM annihilation at the decoupling time while would be irrelevant at present times. Contrary to the NMSSM, the couplings $\lambda _a^b$ and $\lambda _a^X$ are free and then good solutions can be achieved also off-resonance by increasing accordingly this product. Another alternative would to consider, the case $m_X > m_a$. In such a case the DM would feature the additional annihilation channel $X \bar{X} \rightarrow aa$. This channel also features a p-wave annihilation cross section [35, 63], which could again influence the DM relic density without affecting the GC signal. This cross section is, however, relevant for rather light masses of $a$, namely $\lesssim O(1)\,\text{ GeV }$ where there are very strong limits coming from flavor observables as well [64].

The GC excess could be fitted, alternatively, in agreement with the DM relic density, in the case the annihilation channel $X \bar{X} \rightarrow aaa$ is kinematically accessible. The corresponding cross section is, infact, s-wave and could provide an interpretation of the GC signal in terms of the annihilation of a DM with mass of the order of 120 GeV [35]. As already mentioned we are not considering these high values of the DM in this paper.

In the setup depicted the $a$ particles decay mostly invisibly.

At LHC the *a*/*s* particles can be produced through top loops and detected through decay into $2\gamma$. It is, however, not possible to infer prospects of detection from DM phenomenology since some of the relevant couplings are not accessible for DM masses in the range favored by the GC excess. In the numerical example we have proposed, these are anyway rather poor since the $a/s$ mostly decay invisibly.

At a $e^+e^-$ collider, s-channel production is severely suppressed and Higgstrahlung does not operate for gauge singlets unless they mix with $h$ which, for a SM $h$, can only happen for $s$. Since the $a$ can be lighter than the Higgs, it would be possible to detect the invisible decays of the latter into $aa$ pairs. Linear colliders being rather efficient Higgs factories it would be possible to measure $m_a$ with the recoil mass technique.

In the presence of $h$–$s$ mixing there could be detectable effects in the SM Higgs width. In the scenario under consideration, namely the Higgs singlet mostly decaying invisibly, the strongest effect would be in the invisible branching fractions of the Higgs, which are already constrained by LHC limits. We have indeed

16 $$\begin{aligned}&\mathrm{BR}(h \rightarrow \bar{X} X)/\mathrm{BR}(h \rightarrow \bar{b} b)\sim |\lambda _a^X|^2 \tan \alpha ^2/|\lambda _a^b|^2 \end{aligned}$$

where $\alpha$ is the effective mixing angle between $s$ and the Higgs boson. For the numerical example considered above we have $\tan \alpha \lesssim$0.2. Alternatively it is possible to look at deviations of the $hZZ$ coupling from the SM prediction. In our case there would result a suppression by a factor $cos\alpha$. In $e^+e^-$ this coupling is measured to better that $1\,\%$, which corresponds to a mixing angle $\sim$0.1.

## Higgs doublets

### The invisible A scenario

A larger variety of experimental signatures can be provided in a 2 Higgs-doublet scenario. Particularly interesting would be a type II two Higgs-doublet scenario [68] (SUSY models belong to this category). Indeed in this setup we have a $\tan \beta$ enhancement of the coupling of the pseudoscalar with bottom quarks,

17 $$\begin{aligned} \lambda _A^b=\frac{g m_b \tan \beta }{2 m_W}. \end{aligned}$$

The DM annihilation cross section and the decay widths of the $A$ into (Majorana) DM and b quark pairs are

18 $$\begin{aligned}&\langle \sigma v \rangle =\frac{3 |\lambda _A^b \lambda _A^X|^2 s}{32 \pi {\left( s-m_A^2\right) }^2}\nonumber \\&\Gamma (A \rightarrow \bar{b} b)=\frac{|\lambda _A^b|^2 m_A^2}{8\pi },\nonumber \\&\Gamma (A \rightarrow \bar{X} X)=\frac{|\lambda _A^X|^2 m_A^2}{16\pi }. \end{aligned}$$

A value of the annihilation cross section compatible with the GC excess is obtained for $m_A=300$ GeV, $\mathrm{tan}\beta =10$, and $m_X=35$ GeV and $|\lambda _A^X \lambda _a^b|=0.25$, implying $|\lambda _A^X| =2$. With this value, an on mass shell A decays visibly in $\sim 2.5\,\%$ of the cases. In principle, A can also decay into $Zh$ but, for a heavy $A$, the $ZhA$ coupling is too small to contribute significantly. While this solution requires an extended Higgs sector, it satisfies all present constraints. In particular LHC cannot exclude this solution given that A decays invisibly in $>90\,\%$ of the cases. The heavy Higgs boson $H$ is typically almost degenerate in mass with the pseudoscalar. Its main decay channel is into two SM Higgs bosons $h$. This decay mode has been searched at LHC using h decays into two photons and four leptons [69, 70]. This kind of searches applies only for $\tan \beta \sim 1$.

**Fig. 8:**
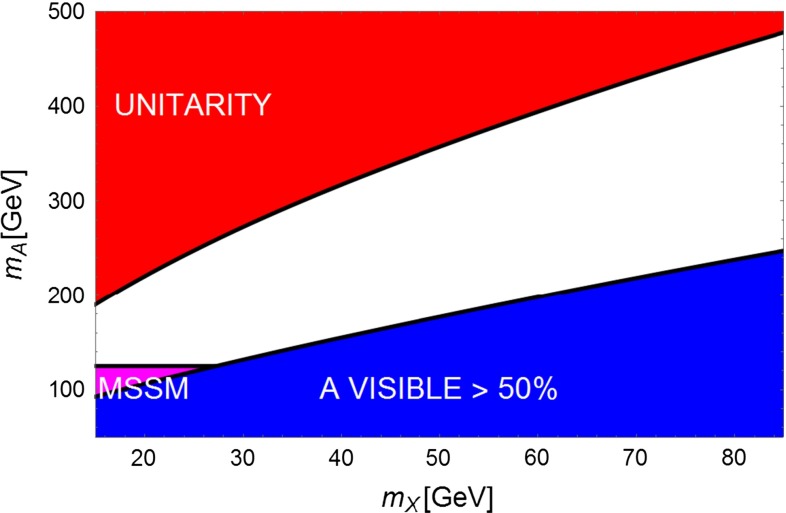
Summary of LHC prospects for $Z^{'}$ for $A$ portal reproducing the GC signal for $\tan \beta =5$. The *blue region* corresponds to visible branching ratio of $A$ greater than $50\,\%$, then within the reach for the LHC. The *magenta region* corresponds to the LHC excluded region in case of a MSSM-like pseudoscalar. The *red region* corresponds to DM coupling above the unitarity limit

**Fig. 9:**
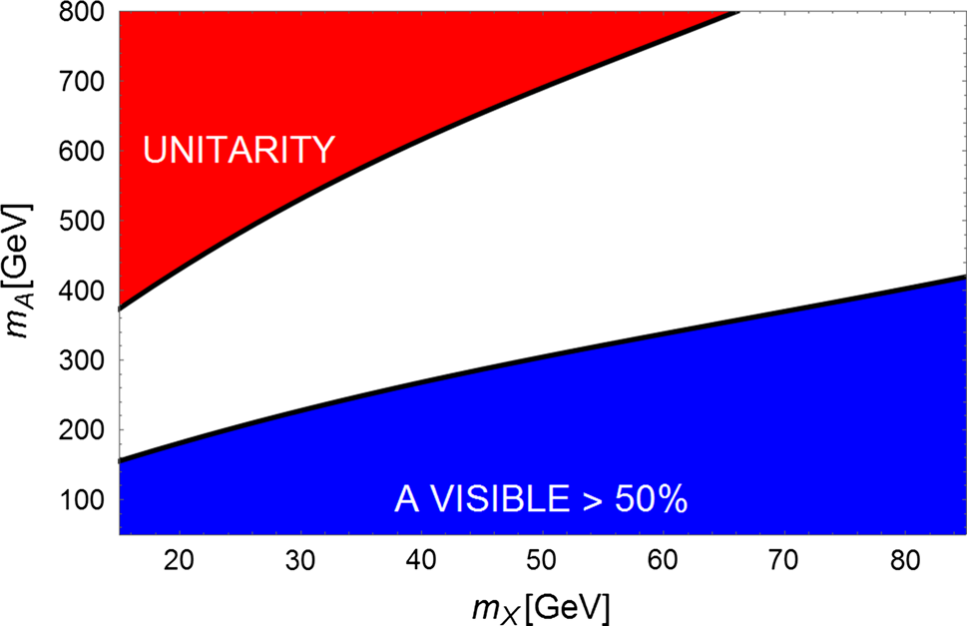
The same as Fig. 9 but for $\tan \beta =20$

Figures 8 and 9 display the mass domain expected for this type of solution. The channel $$HA$$ would be accessible to a TeV $$e^+e^-$$ collider provided that $$m_A<500$$ GeV. It would allow one to tag the presence of invisible decays of A by using a recoil mass technique by reconstructing the accompanying H boson. Typically, for an integrated luminosity of $$1 ab^{-1}$$, one expects $$\sim 7 000$$$$HA$$ events [71] with $$A$$ decaying mostly invisibly.

For what concerns H, which will serve to tag the presence of an invisible A, the standard decay mode is dominated by hh $(98\,\%)$. ILC detectors are optimized to perform this type of analysis with precise jet energy measurement ($3\,\%$ resolution). One can use heavy quark identification for $h$ decaying predominantly into pairs of $b$ quarks. The main background, which comes from top pairs producing only two $b$ jets, is easily rejected.

**Fig. 10:**
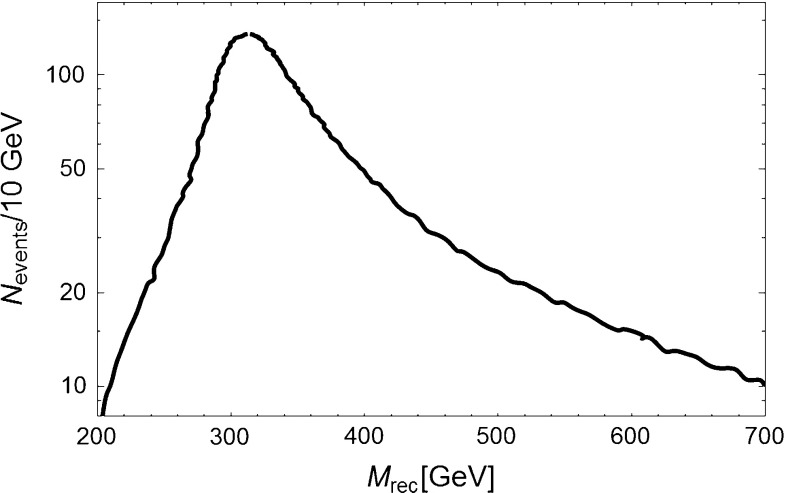
Predicted recoil mass distribution using the H decay into 4b for a $A$ portal model with $\tan \beta =5$ and $m_A=300\,\text{ GeV }$. The bump corresponds to A decaying invisibly in the HA final state for ECM=1 TeV and with a luminosity of $1 ab^{-1}$

Figure 10 shows the expected recoil mass distribution obtained using, for the $HA$ channel at $E_\mathrm{CM}=1$ TeV, a realistic energy resolution for the H decaying into 4b (from the 2h) and including initial state radiation. The A resonance parameters can be precisely measured with $m_A=300 \pm 0.8$ GeV and $\Gamma _A=24\pm 1$ GeV. From the latter, one can extract the coupling with $2\,\%$ accuracy.

### The invisible A scenario at LHC

In the case of $A$ substantially decaying invisibly the most constraining limits come from searches of $H^{\pm }$ decays [72–74] which can be translated into the limit $m_A>140$ GeV, assuming a MSSM-like spectrum for the extra Higgs bosons. As can be seen from Fig. 8, the GC excess solution with $m_X=35$ GeV corresponds to $m_A >150$ GeV, which is not excluded by LHC.

**Fig. 11:**
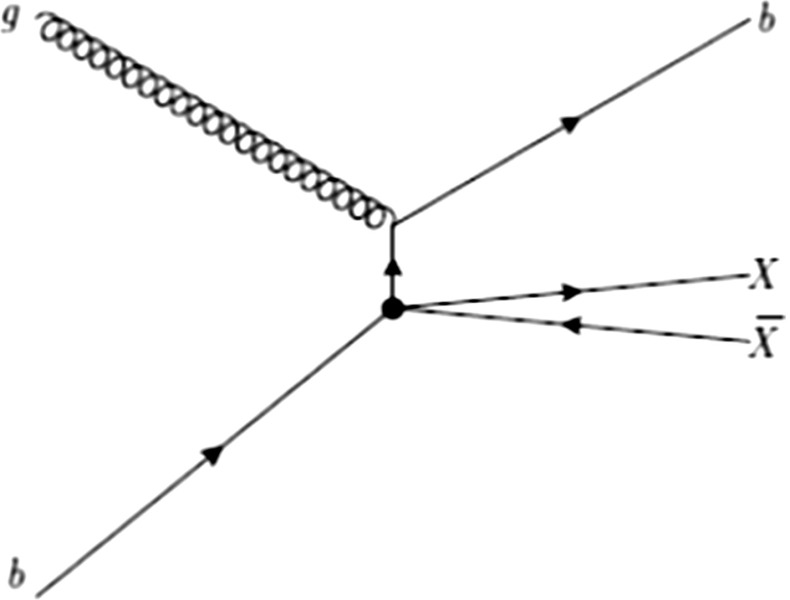
Diagram relative to the DM pair production process in association with a b-jet as proposed in [75]

An alternative detection strategy for an invisible $A$ has been suggested in [75]. The relevant process is represented in Fig. 11: a gluon scatters a b quark from the sea, which radiates a A boson decaying into DM. The monojet in this case is a b-jet which allows one to tag this mechanism. Reference [76] indicates that the required sensitivity is still way below what is needed to observe this signal. This sensitivity depends on the coupling of A to b quarks which is proportional to $\tan \beta$. Scenarios like the one under consideration require 14 TeV center of mass energy and $40 {\text{ fb }}^{-1}$ integrated luminosity to be probed. While these prospects appear promising, it will be difficult to interpret unambiguously the origin of an excess of monojet production, for instance as due to A or to a $Z^{'}$. One may, of course, hope that other signals due to a two-doublet scenario will give direction as to the interpretation.

## Conclusions

We have considered some simple DM scenarios, possibly providing an interpretation of the recently reported $\gamma$-ray excess from the GC. A fermionic DM has been assumed to interact with two SM mediators, the $Z$ and $h$ bosons, and two BSM mediators, the $Z^{'}$ and a pseudoscalar mediator. We have, in particular, focused on the discovery prospects of $e^+e^-$ colliders compared with current LHC searches.

In the case of $Z$ boson mediator, Direct Detection limits force to require substantially pure axial coupling with the DM. Although this implies a DM annihilating mostly into $\bar{b} b$ it is not possible to reproduce the GC excess, unless extra effects like the Sommerfeld enhancement are invoked, since the velocity dependence of the annihilation cross section implies a strong mismatch between the present time and decoupling values. The invisible *Z* width is the most sensitive SM observable to monitor this scenario. With the accuracy given by LEP1, one can already disfavor $m_X <27$ GeV. Future $e^+e^-$ colliders will reach $m_X <35$ GeV.

$Z{'}$ scenarios allow one to reproduce the GC excess, evading at the same time DD constraints, due to a greater freedom in the choice of the parameters. This kind of result is achieved, for example, for a $Z^{'}$ with pure vectorial coupling of the DM and pure axial couplings with SM fermions. These last couplings should be rather suppressed, in order to satisfy the LHC limits on dilepton searches, implying a $Z^{'}$ decaying mostly invisibly. This $Z^{'}$ is accessible to detection at a TeV $e^+e^-$ collider through radiative return. This technique allows one to observe the $Z^{'}$ resonance and determine its mass, width and coupling to $e^+e^-$. Generally speaking, the ISR technique in $e^+e^-$ provides a powerful tool to detect DM, provided that one can run this collider with highly polarized beams to eliminate the $e^+e^- \rightarrow \nu _e \nu _e \gamma$ process due to $W$ exchange. It also requires an optimized setup to fully eliminate the contamination from $e^+e^- \rightarrow e^+e^- \gamma$.

In the case of scalar mediators the SM Higgs is ruled out by limits from DM Direct Detection and invisible decay width of the Higgs itself.

Interactions mediated by a pseudoscalar Higgs single state are a rather simple and economic way to account for the GC excess. This state can be lighter than the SM Higgs boson and then observed in its decays. Associate production of this particle with a $Z$ boson is observable in a $e^+e^-$ collider down to a high sensitivity.

A larger variety of collider signatures in the case the pseudoscalar state is achievable in a two-doublet Higgs extension of the SM. Moreover, in this kind of scenarios it is possible to naturally achieve a DM mostly annihilating into bottom pairs. In this kind of scenario the DM is assumed to couple exclusively with the pseudoscalar component of the Higgs spectrum which decays mostly invisibly. It can be observed at a TeV $e^+e^-$ collider in associated production with the heavy scalar boson $H$ and its mass and decay width can be measured with high precision.

The interpretation of the GC excess seems to disfavor SM particles as mediators of the DM interaction. If this excess would be confirmed it would provide an indication of the existence of extra particles, besides the DM, beyond the SM. Although several scenarios can already tested at LHC, a TeV $e^+e^-$ collider would provide an essential tool for a precise measurement of the relevant parameters.

**Note added:** Soon after the completion of this work new analyses [77, 78] have shown that the GC excess is compatible with a broader range of DM masses and final state annihilation channels. These results are especially relevant in scenarios of Higgs mediators. Contemporary other possible $Z^{'}$ scenarios accounting for the GC excess have been presented in [79].

## Footnotes

1 Conscious that an UV realization of this kind of framework could be challenging, we keep our analysis at the low energy level.
